# RecC^935H^ is associated with divergent evolutions of clonal group 258 *Klebsiella pneumoniae*

**DOI:** 10.1080/21505594.2026.2673646

**Published:** 2026-05-13

**Authors:** Dakang Hu, Xinru Ye, Jie Wang, Shuli Mi, Jiawen Sun, Piaopiao Dai, Tingting Huang, Jin Zhang, Xinhua Luo, Qinfei Ma, Xiaofei Jiang, Susu Wu, Haifang Zhang

**Affiliations:** a Department of Laboratory Medicine, Taizhou Municipal Hospital (Taizhou University Affiliated Municipal Hospital), School of Medicine, Taizhou University; Taizhou Key Laboratory of Infection and Tumor Immunology; Key Laboratory of Sepsis of Taizhou, Taizhou, China; b Medical College, Taizhou University, Taizhou, China; c Premium Clinic, Huashan Hospital, Fudan University, Shanghai, China; d Department of Laboratory Medicine, Taizhou Municipal Hospital (Taizhou University Affiliated Municipal Hospital), School of Medicine, Taizhou University, Taizhou, China; e Department of Medical Laboratory, Taizhou Traditional Chinese Medicine Hospital, Taizhou, China; f Department of Medical Laboratory, Huashan Hospital, Fudan University, Shanghai, China; g Department of Clinical Laboratory, The Second Affiliated Hospital of Soochow University, Suzhou, Jiangsu, China; h Department of Laboratory Medicine, Taizhou Municipal Hospital (Taizhou University Affiliated Municipal Hospital), School of Medicine, Taizhou University, Taizhou Key Laboratory of Infection and Tumor Immunology, Taizhou, China; i MOE Key Laboratory of Geriatric Diseases and Immunology, Soochow University, Suzhou, Jiangsu, China

**Keywords:** *Klebsiella pneumoniae*, drug resistance, virulence, sequence type, evolution

## Abstract

Clonal group 258 *Klebsiella pneumoniae* strains are notorious but their divergent evolutions were identified through analysis of 80 sequence type (ST) 258, 284 ST11, and 15 ST512 genomes. The serotypes of ST11 strains differed from those of ST258 and ST512. Genes *bla*_KPC-2_ and *bla*_KPC-3_ dominated carbapenem-resistance in ST258 strains, whereas they were found in ST11 and ST512, respectively. The carbapenem-resistance rates were 93.75%, 86.97%, and 100.0% among the ST258, ST11, and ST512 strains, respectively while the hypervirulence and carbapenem-resistance rates were 2.50%, 35.92%, and 0.00%, respectively. The wide carbapenem-resistance and the hypervirulence were determined by drug-resistance and virulence plasmids respectively. Except *recA*, *recB*, and *recD*, *recC* was significantly different: RecC^935R^ for the three groups and RecC^935H^ for ST11; RecC^935H^ ST11 strains presented a higher rate of hypervirulence plus carbapenem-resistance than those carrying RecC^935R^. RecC^935H^ and RecC^935R^ presented the difference of an amino acid side chain, leading to the disappearance of the hydrogen bond. Single-Nucleotide Polymorphism analysis verified closer relationship between ST258 and ST512 than ST11. RecC^R935H^ facilitated pK2044 to HS11286 (5.61 times) while *wzy-K1* deletion exerted advantages on pKPHS2 to NTUH-K2044 (5.49 times). The retention rates of pK2044 in HS11286^*recC*2804G^+*arr-3* kept over 80.0% in the 8 passages while those in HS11286^*recC*2804A^+*arr-3* declined to less than 3.0% in the last 5 passages. In conclusion, different from ST258 and ST512, ST11 strains show an overwhelming propensity to become hypervirulent and carbapenem-resistant. RecC^R935H^ mutation facilitates the transfer of virulence plasmids into carbapenem-resistant *K. pneumoniae* strains but decreases their retention in the strains.

## Introduction

*Klebsiella pneumoniae*, a prominent member of the family *Enterobacteriales*, is capable of causing infections at virtually any anatomical site in humans [1], contributing to substantial clinical mortality. For instance, the 28-day mortality rate associated with *K. pneumoniae* bacteremic pneumonia reaches 52% [2]. As a key member of the “ESKAPE” pathogens (*Enterococcus faecium*, *Staphylococcus aureus*, *Klebsiella pneumoniae*, *Acinetobacter baumannii*, *Pseudomonas aeruginosa*, and *Enterobacter species*) [3], *K. pneumoniae* has drawn global attention due to its escalating antimicrobial resistance. With widespread antibiotic usage, over 100 distinct antibiotic-resistance genes have been identified in this species [4], among which carbapenemase genes—conferring resistance to carbapenems—are of particular and sustained concern. Carbapenemases, including KPC, NDM, IMP, VIM, and OXA-48, hydrolyze carbapenems to mediate resistance [5]. Additionally, reduced expression or loss of the outer membrane porins OmpK35 or OmpK36 can further impair carbapenem susceptibility by blocking their intracellular transport [6,7]. As major porins in *K. pneumoniae*, OmpK35 and OmpK36 share homology with *Escherichia coli*’s OmpF and OmpC, respectively [8]. These proteins are critical for bacterial survival: they facilitate the influx of essential compounds (e.g. nutrients and antimicrobials) and, together with peptidoglycans and lipopolysaccharides, maintain cell envelope integrity [9,10].

Clinically, carbapenem-resistant *K. pneumoniae* (CRKP) is more prevalent in nosocomial pneumonia (58.2%) than in community-acquired pneumonia (CAP; 5.8%), whereas hypervirulent *K. pneumoniae* (HvKP) shows the opposite trend—predominating in CAP (61.5%) over nosocomial infections (16.3%) [2]. CRKP accounts for approximately 25.0% of clinical *K. pneumoniae* isolates globally [11], while hypervirulent carbapenem-resistant *K. pneumoniae* (Hv-CRKP), though emerging, constitutes a growing subset (5.0%) [12].

Mortality data underscore the severity of CRKP infections: pooled analysis revealed a 42.14% mortality rate for CRKP infections versus 21.16% for carbapenem-susceptible *K. pneumoniae* (CSKP) [13]. Geographically, CRKP mortality varies but remains elevated: 33.24% (North America), 46.71% (South America), 50.06% (Europe), and 44.82% (Asia) [13], doubling the mortality rate of CSKP. While robust large-scale studies are limited, Hv-CRKP infections exhibit even higher fatality. For example, Hv-CRKP-induced meningitis carried a 92.3% mortality rate (12/13) compared to 56.5% (13/23) for non-Hv-CRKP (*p* < 0.05) [14]; another report documented a 5/9 fatality in postoperative Hv-CRKP meningitis versus 1/11 in non-Hv-CRKP cases [15]. Additionally, a study of five Hv-CRKP strains causing severe pneumonia reported 100% mortality [16].

Most CRKP strains belong to clonal group (CG) 258, which encompasses sequence types (STs) 258, 11, and 512 [17]. Less common but documented members include ST270, ST340, ST379, ST407, and ST418 [18]. CG258 is classified as a global high-risk clone due to its: (i) wide geographic distribution; (ii) multidrug resistance profile; (iii) enhanced fitness, virulence, and pathogenicity; (iv) prolonged host colonization and persistence; and (v) efficient interhost transmission [19]. Notably, ST258 CRKP dominates in Western countries [20,21], whereas ST11 CRKP is endemic in East Asia, particularly China [22]. The drivers of this geographic disparity remain poorly understood. To address this knowledge gap, we analyzed 80 ST258, 284 ST11, and 15 ST512 genomes (collected from GenBank due to challenges in concurrent isolation of ST258 and ST11 strains) to characterize differences in serotypes, antibiotic-resistance genes, outer membrane protein-related genes, virulence genes, and the RecBCD/A system. Furthermore, we investigated the impact of the RecC^935H^ mutation on virulence plasmid transfer efficiency and plasmid retention using mating assays and plasmid retention tests.

## Methods

### *K. pneumoniae* strains

A total of 1,407 *K. pneumoniae* genomes (Table S1) were retrieved from GenBank (https://www.ncbi.nlm.nih.gov/datasets/genome/?Taxon=573; accessed 30 August 2022). Their chromosomal accession numbers were submitted to the Multilocus Sequence Typing (MLST) database (https://bigsdb.pasteur.fr/cgi-bin/bigsdb/bigsdb.pl?db=pubmlst_klebsiella_seqdef&page=sequenceQuery) to determine their STs. From this collection, 379 strains belonging to the CG258 group (Table S2) were selected for subsequent analyses, comprising 80 ST258, 284 ST11, and 15 ST512 strains. Strains of other STs (excluding ST340) were excluded due to their limited representation (fewer than 10 strains each), and 22 ST340 strains were further omitted because their serotypes could not be reliably predicted.

The 80 ST258 strains originated from diverse geographic regions: the United States (52 strains), Japan (15), Australia (6), the United Kingdom (3), France (1), Italy (1), Colombia (1), and Switzerland (1). The 284 ST11 strains were primarily from East Asia, with the majority originating from mainland China (208 strains), followed by Taiwan (15), the United States (14), Switzerland (12), Hong Kong (10), the Czech Republic (4), India (4), Germany (3), Russia (3), Japan (3), Spain (3), Australia (1), Canada (1), Italy (1), the United Kingdom (1), and Norway (1). The 15 ST512 strains were distributed across Italy (7), the United States (3), Switzerland (2), the Czech Republic (1), Germany (1), and mainland China (1).

### Determination of serotypes, antibiotic-resistance, and virulence genes

The chromosomal accession numbers of these 379 strains were submitted to the Institute Pasteur database (https://bigsdb.pasteur.fr/cgi-bin/bigsdb/bigsdb.pl?db=pubmlst_klebsiella_seqdef&page= sequenceQuery) to predict their serotypes. For strains with untypable serotypes, conventional PCR primers targeting K47 and K64 were employed for detection [23].

To identify antibiotic-resistance genes (including *bla*_KPC_, *bla*_KPC-2_, *bla*_KPC-3_, *bla*_KPC-12_, *bla*_KPC-31_, *bla*_VIM_, *bla*_IMP_, *bla*_NDM_, and *bla*_OXA-48_), the 379 genome sequences were analyzed using the ResFinder tool (https://cge.food.dtu.dk/services/ResFinder/).

Virulence gene prediction (encompassing *peg-344*, *allS*, *rcsA*, *rcsB*, *p-rmpA*, *p-rmpA2*, *c-rpmA*, *wza*, *wzb*, *wzi*, *fimH*, *mrkD*, *entB*, *irp2*, *iroN*, and *iucA*) was performed via the Virulence Factors of Pathogenic Bacteria database (http://www.mgc.ac.cn/cgi-bin/VFs/v5/main.cgi). These virulence genes were used to infer specific virulence traits: *allS* (allantoin metabolism), *p-rmpA/p-rmpA2/c-rpmA* (hypercapsule synthesis), *wzi* (capsule existence), *entB* (enterobactin production), *irp2* (yersiniabactin biosynthesis), *iroN* (salmochelin synthesis), *iucA* (aerobactin synthesis), *fimH* (type 1 fimbriae), and *mrkD* (type 3 fimbriae).

Outer membrane protein (OMP)-related genes (*ompK35*, *ompK36*, *ompK26*, *ompK37*, *ompR*, *ompA*, *lamb*, *kbvR*, *envZ*, and *ramA*) were predicted using NCBI BLAST (https://blast.ncbi.nlm.nih.gov/Blast.cgi?PROGRAM=blastn&PAGE_TYPE=BlastSearch&LINK_LOC=blasthome) with experimental validation (Table S3). A cutoff of 80% sequence coverage and 80% identity was applied for homology matching. Reference sequences for these OMP genes were derived from the genome of *K. pneumoniae* SGH10 (Accession No.: CP025080.1) [24].

### Definition of CRKP, HvKP, and Hv-CRKP

CRKP was defined by the presence of *bla*_KPC_, *bla*_VIM_, *bla*_IMP_, *bla*_NDM_, or *bla*_OXA-48_ or the absence of *ompK35* or *ompK36*. HvKP was used as a reference [25]. However, the capsule is essential for hypervirulence [26]. Therefore, HvKP has a positive *wzi*. Hv-CRKP was defined as both CRKP and HvKP.

### Construction of phylogenetic tree

The MEGA-X [27] was used to build phylogenetic trees of *recA*, *recB*, *recC*, *recD*, and RecC from the 379 *K. pneumoniae* strains using the maximum likelihood and neighbor-joining methods. One thousand bootstrap replicates were used.

The tool kSNP4 [28] was used to identify single-nucleotide polymorphism (SNP) between all our selected genomes without needing a reference genome. The construction of a SNP-based phylogenetic tree using the maximum likelihood method revealed the clusters of core genomes.

### Modeling of RecC

The models of RecBCD enzyme subunit RecC were built by AlphaFold 2.

### Construction of NTUH-K2044 and HS11286 mutants

*K. pneumoniae* NTUH-K2044 was isolated from the Department of Internal Medicine, National Taiwan University Hospital (Taipei, Taiwan). This strain was serotyped as K1 and genotyped as ST23, and carried the virulence plasmid pK2044. A Δ*wzy-K1* mutant (NTUH-K2044Δ*wzy-K1*) was generated as a reference strain [29] and has also been utilized in a prior study [30]. Compared to the wild-type NTUH-K2044, which exhibited a hypercapsular phenotype, the Δ*wzy-K1* mutant displayed a slim capsule structure [30]. Both NTUH-K2044 and NTUH-K2044Δ*wzy-K1* showed resistance to ampicillin and potassium tellurite.

*K. pneumoniae* HS11286 (Accession No.: CP003200.1) was isolated from the Department of Laboratory Medicine, Huashan Hospital, Fudan University (Shanghai, China). This strain was serotyped as K47 and genotyped as ST11, with the carbapenemase gene *bla*_KPC-2_ located on the plasmid pKPHS2. Two *recC* mutants—HS11286^*recC*2804G^+*arr-3* and HS11286^*recC*2804A^+*arr-3* were constructed using λ-Red recombination method [29], with the *arr-3* gene inserted at downstream of *recC*. Both mutants displayed resistance to meropenem and rifampicin.

Primer sequences used in this study are listed in Table S4. All three mutants (NTUH-K2044Δ*wzy-K1*, HS11286^*recC*2804G^+*arr-3*, and HS11286^*recC*2804A^+*arr-3*) were validated through conventional PCR amplification, gel electrophoresis, and Sanger sequencing.

### Mating tests

In the first-round conjugation assays, *K. pneumoniae* HS11286^*recC*2804G^+*arr-3* and HS11286^*recC*2804A^+*arr-3* served as donors, while NTUH-K2044 and NTUH-K2044Δ*wzy-K1* acted as recipients. Donor and recipient strains were first cultured overnight, then subcultured to mid-logarithmic growth phase. Cell mixtures were prepared by combining 200 μL of donor cells with 800 μL of recipient cells, followed by centrifugation at 10,000 × g for 1 min. The resulting pellet was washed once with 1 mL of normal saline (NS) and recentrifuged under the same conditions. Forty percents of the resuspended pellet (50 μL) were then spotted onto a filter membrane placed on a sheep blood agar plate. After an overnight incubation, the bacterial lawn was harvested by washing with NS, and transconjugants were selected on Luria-Bertani (LB) agar plates supplemented with 1.5 μg/mL potassium tellurite and 2.0 μg/mL meropenem. Putative transconjugants (NTUH-K2044+pKPHS2 and NTUH-K2044Δ*wzy-K1*+pKPHS2) were validated via successful PCR amplification of *p-rmpA2* (from pK2044) and *bla*_KPC-2_ (from pKPHS2).

In the second-round conjugation experiments, HS11286^*recC*2804G^+*arr-3* and HS11286^*recC*2804A^+*arr-3* were used as recipients, whereas NTUH-K2044 +pKPHS2 and NTUH-K2044Δ*wzy-K1*+pKPHS2 served as donors. Following the same growth protocol (overnight culture followed by subculture to mid-log phase), 8 mL of donor cells were mixed with 2 mL of recipient cells. The mixture was centrifuged at 10,000 × g for 1 min, and the pellet was washed once with 10 mL of NS. After re-centrifugation, the resuspended pellet (50 μL) was spotted onto a filter membrane on sheep blood agar. Overnight incubation was followed by lawn harvesting with NS, and transconjugants were selected on LB agar plates containing 1.5 μg/mL potassium tellurite and 30 μg/mL rifampicin. Candidate transconjugants (HS11286^*recC*2804G^+*arr-3*+pK2044 and HS11286^*recC*2804A^+*arr-3*+pK2044) were confirmed by PCR amplification of *p-rmpA2* (from pK2044) and *arr-3*.

All mating assays were performed in triplicate to ensure reproducibility.

### Virulence plasmid retention tests

To assess plasmid retention, *K. pneumoniae* strains HS11286^*recC*2804G^+*arr-3*+pK2044 and HS11286^*recC*2804A^+*arr-3*+pK2044 were cultured in LB broth at 30°C with subculturing every 12 hours for a single passage. The resulting cell suspensions were serially diluted to 10^9^ folds using NS, and 100 μL of each dilution was plated onto both nonselective LB agar plates and selective LB agar plates supplemented with 1.5 μg/mL potassium tellurite and 30 μg/mL rifampicin. The retention ratio of pK2044 was calculated as the ratio of colony-forming units (CFUs) recovered on selective LB agar to those on nonselective LB agar. This assay was repeated over 8 consecutive passages to evaluate plasmid stability; it was done in triplicate in every passage.

### Statistical analysis

Statistical analyses were performed using GraphPad Prism 8 software (GraphPad Software Inc., CA, USA). Chi-square test, Fisher’s exact test, and Wilcoxon test were used for comparisons between groups. Statistical significance was set at a *p* value less than 0.05.

## Results

### Prevalence of serotypes, resistance, and virulence genes in ST258, ST11, and ST512 strains

**Figure 1.**
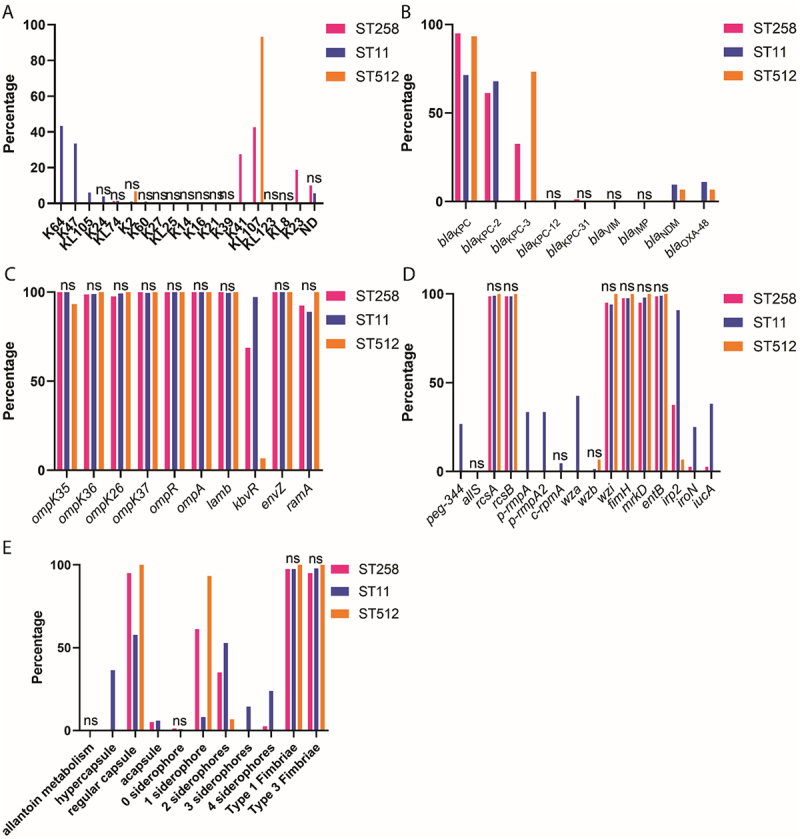
Distribution of serotypes, drug-resistance genes, outer membrane protein-related genes, virulence genes, and virulence factors among ST258, ST11, and ST512 *K. pneumoniae* strains; (A) Distribution of serotypes among ST258, ST11, and ST512 strains; (B) Prevalence of various *bla* genes among ST258, ST11, and ST512 strains; (C) Prevalence of outer membrane protein-encoding genes and their regulators among ST258, ST11, and ST512 strains; (D) Prevalence of 16 virulence genes among ST258, ST11, and ST512 strains; (E) Prevalence of predicted virulence factors among ST258, ST11, and ST512 strains.

ST, sequence type; ns, no significant difference; ND, not defined.

A total of 18 distinct serotypes were identified among the 379 *K. pneumoniae* strains (Figure 1(A)). Within ST258, KL107 (34/80) and K41 (22/80) were the most prevalent serotypes, with KL107 (14/15) exclusively dominating ST512. ST11 strains primarily harbored K64 (123/284) and K47 (95/284). Notably, no serotype was shared between ST258 and ST11: ST258 strains exhibited only three serotypes (KL107, K41, and K23), whereas ST11 presented 12 serotypes, including K64 and K47.

Although carbapenemase gene (*bla*_KPC_) was predominant across all three ST groups, its prevalence was higher in ST258 and ST512 than in ST11 (Figure 1(B)). In ST258, *bla*_KPC-2_ and *bla*_KPC-3_ accounted for all *bla*_KPC_ variants. ST512 strains predominantly harbored *bla*_KPC-3_, whereas ST11 strains primarily carried *bla*_KPC-2_. Except for *kbvR*, all other OMP-related genes were ubiquitously distributed among the three groups (Figure 1(C)). ST11 exhibited a significantly higher prevalence of *kbvR* (97.18%) compared to ST258 (68.75%) and ST512 (6.67%).

Virulence genes *rcsA*, *rcsB*, *fimH*, *mrkD*, and *entB* were highly prevalent (≥95%) in all groups (Figure 1(D)). ST11 strains showed higher positive rates for *peg-344*, *p-rmpA*, *p-rmpA2*, *wza*, *irp2*, *iroN*, and *iucA* than both ST258 and ST512. With the exception of *irp2* (which showed un-comparable prevalence between ST258 and ST512), all other virulence genes exhibited equal positive rates between ST258 and ST512 (Figure 1(D)). Notably, compared to the “regular” capsule phenotype, ST11 strains displayed a higher frequency of hypercapsule production and carried 2–4 siderophores, whereas ST258 and ST512 showed lower rates of these traits (Figure 1(E)).

### Prevalence and determinants of CRKP, HvKP, and Hv-CRKP in ST258, ST11, and ST512 strains

**Figure 2.**
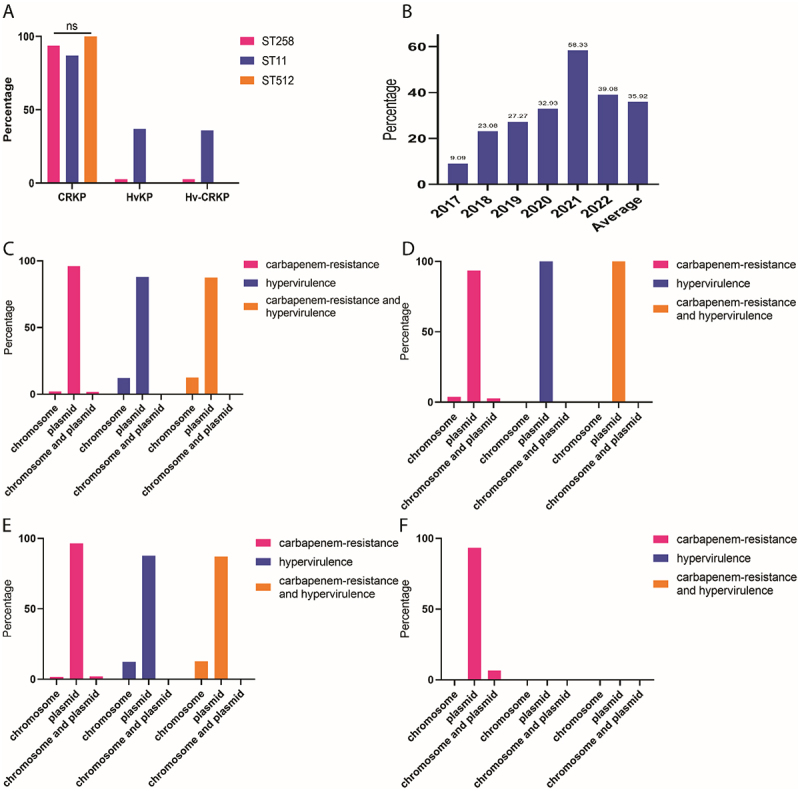
Prevalence of CRKP, HvKP, and Hv-CRKP among ST258, ST11, and ST512 *K. pneumoniae* strains and rates of Hv-CRKP in ST11 strains in different years; (A) Prevalence of CRKP, HvKP, and Hv-CRKP among ST258, ST11, and ST512 strains; (B) Prevalence of Hv-CRKP in ST11 strains in different years; (C) Vectors of carbapenem-resistance, hypervirulence, and carbapenem-resistance plus hypervirulence among the 379 *K. pneumoniae* strains; (D) Vectors of carbapenem-resistance, hypervirulence, and carbapenem-resistance plus hypervirulence in 80 ST258 strains; (E) Vectors of carbapenem-resistance, hypervirulence, and carbapenem-resistance plus hypervirulence in 280 ST11 strains; (F) Vectors of carbapenem-resistance, hypervirulence, and carbapenem-resistance plus hypervirulence in 15 ST512 strains.

ST: sequence type; ns: no significant difference; CRKP: carbapenem-resistant *Klebsiella pneumoniae*; HvKP: hypervirulent *Klebsiella pneumoniae*; Hv-CRKP: hypervirulent carbapenem-resistant *Klebsiella pneumoniae*; Average indicates the average rates between 2017 and 2022.

Although the CRKP rates were comparable across the three CGs (ST258, ST11, and ST512; all >86.00%), ST11 strains exhibited significantly higher proportions of HvKP and Hv-CRKP. Notably, both HvKP and Hv-CRKP rates were <3.00% in ST258 and ST512 (Figure 2(A)). Within ST11, the Hv-CRKP rate increased from 9.09% (2017) to 58.33% (2021), then declined to 39.08% (2022), with an overall average of 35.92% (Figure 2(B)). Prior to 2017, no Hv-CRKP cases were detected. Carbapenem resistance was primarily mediated by plasmids (>93.0%; Figure 2(C–F)), while hypervirulence was also largely driven by plasmids (>87.0%; Figure 2(C–E)).

### RecBCD/A system in *K.* *pneumoniae* strains of ST258, ST11, and ST512

**Figure 3.**
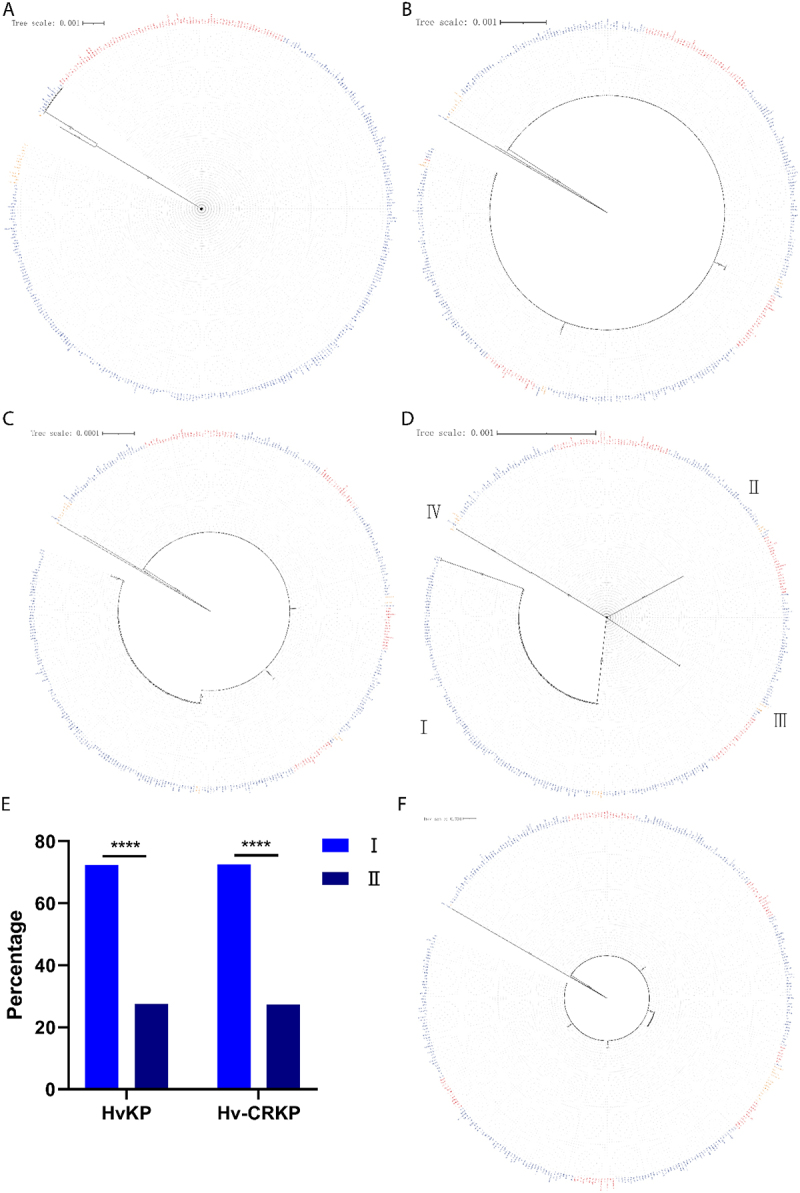
Prevalence of RecABCD systems among ST258, ST11, and ST512 *K. pneumoniae* strains; (A) Phylogenic tree of *recA* among ST258, ST11, and ST512 strains; (B) Phylogenic tree of *recB* among ST258, ST11, and ST512 strains; (C) Phylogenic tree of *recC* among ST258, ST11, and ST512 strains; (D) Phylogenic tree of RecC among ST258, ST11, and ST512 strains; (E) Prevalence of HvKP and Hv-CRKP in clade I and II ST11 strains; (F) Phylogenic tree of *recD* among ST258, ST11, and ST512 strains ns, no significant difference; ****, *p* < 0.0001.

To investigate the underlying causes of the distinct phenotypic traits observed among ST258, ST11, and ST512 *K. pneumoniae* strains, we analyzed their RecBCD/A systems—key components involved in DNA repair and recombination. Sequence alignment revealed that *recA* alleles were nearly identical across all strains, with exceptions limited to 12 ST11 and 1 ST512 strains (Figure 3(A)). Similarly, *recB* sequences showed high conservation, with discrepancies restricted to a small number of strains across the three groups (Figure 3(B)). In contrast, *recC* exhibited notable variation among the groups (Figure 3(C)). Further comparison of RecC proteins identified two distinct allelic variants: one (predominant in ST258/ST512 and over half of ST11 strains, designated Clade II) carried the RecC^935R^ (molecular weight: 128,879.35 Da), while the other (enriched in Clade I) contained RecC^935H^ (molecular weight: 128,860.30 Da). The RecC_R935H_ variant correlated with the *recC*^G<A^ polymorphism. Notably, Clade I strains exhibited a significantly higher rate of Hv-CRKP than Clade II strains (*p* < 0.0001; Figure 3(E)), a difference that persisted even when restricting analysis to CRKP isolates (68/98 vs. 26/95; *p* < 0.0001). *recD* sequences were largely conserved across all three groups, with minor variations limited to a subset of ST11 strains (Figure 3(F)).

### RecC structural analysis and SNP correlations in ST258, ST11, and ST512 *K.* *pneumoniae*

**Figure 4.**
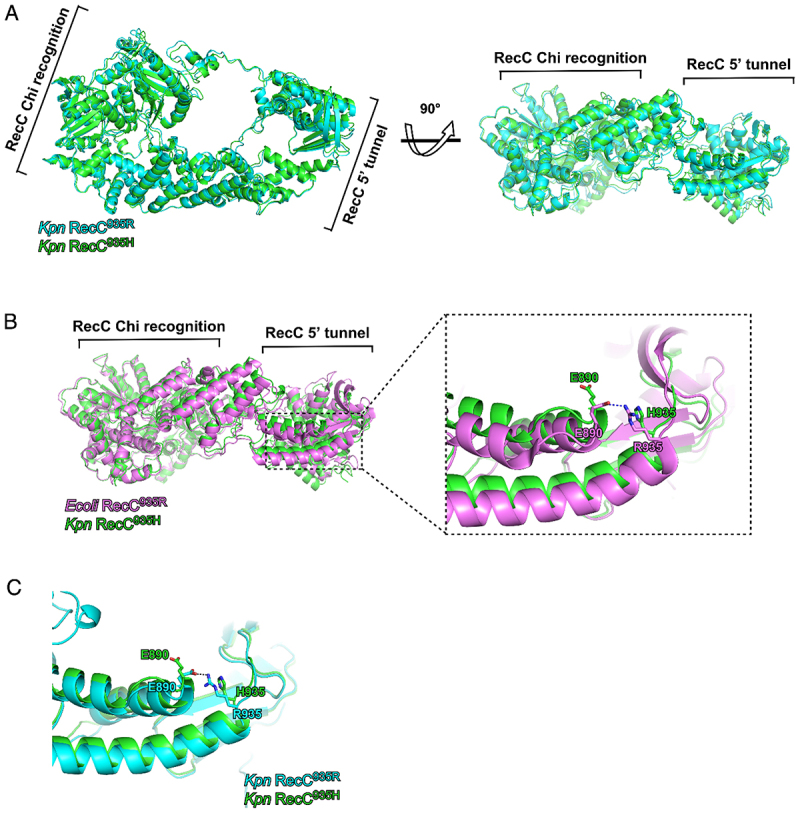
Comparison between the models of RecC935H and RecC935R. (A) the overall models of *Klebsiella pneumoniae* RecC^935H^ and RecC^935R^ were displayed in cartoon mode. Cyan, RecC^935R^. Green, RecC^935H^. (B) the detail comparison of *E.Coli* RecC (PDB code: 6SJF) and *K. pneumoniae* RecC^935H^. Essential residues 935 H and 935 R were showed as sticks. Magenta, *E.Coli* RecC. Dash, hydrogen bond. (C) the detail comparison of *K. pneumoniae* RecC^935H^ and RecC^935R^. *Kpn*: *Klebsiella pneumoniae*; *Ecoli*: *Escherichia coli*.

Structural modeling demonstrated that the RecC proteins of the two mutant variants (RecC^935R^ and RecC^935H^) were nearly identical in overall architecture, with only subtle shifts observed in the 5’ channel domain (Figure 4(A)). Comparative analysis between *K. pneumoniae* RecC^935H^ and *E. coli* RecC revealed that the R935H substitution disrupted the hydrogen bond between residues R935 and E890; histidine (H) cannot form a stable hydrogen bond with glutamate (E) (Figure 4(B)), leading to structural displacement of the 5’ channel domain. In contrast, the R935 residue in wild-type RecC^935R^ maintained a canonical hydrogen bond with E890 (Figure 4(C)).

**Figure 5.**
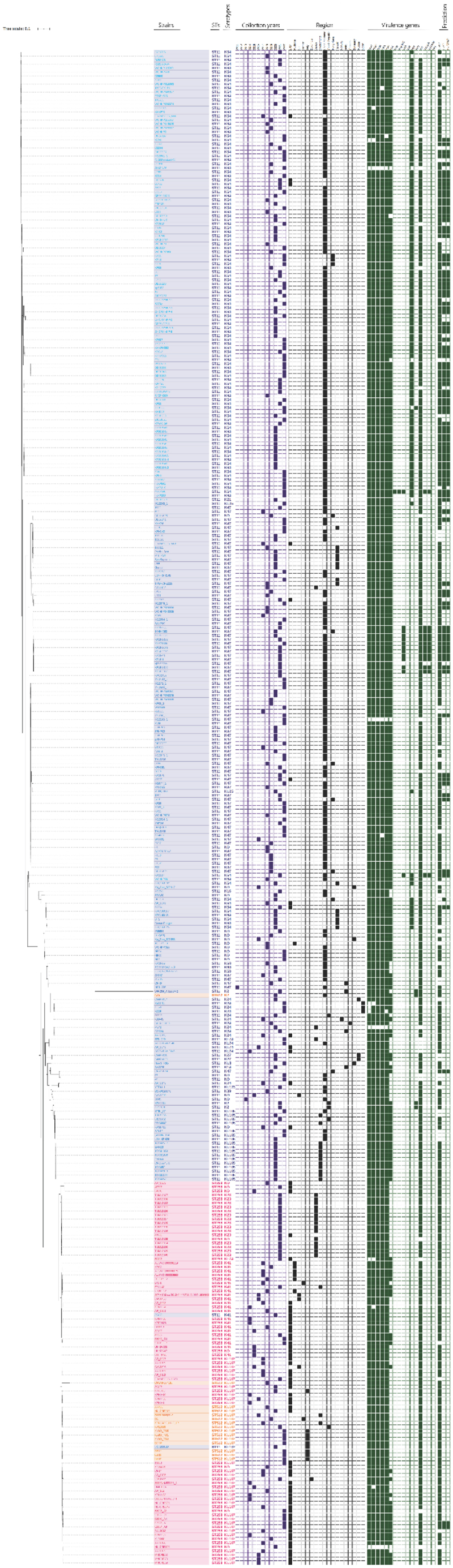
Phylogenetic tree of the 379 strains. The 379 chromosomes were analyzed using kSNP4. SNPs lines represent different strains: light blue for RecC^935H^ ST11 strains, dark blue for RecC^935R^ ST11 strains, and darkest blue for the rest ST11 strains; red for ST258 strains and yellow for ST512 strains. Based on the predicted results, the binary gene presence/absence matrix was created reflecting the collection year, collection region, virulence genes; and prediction of HvKP, CRKP, and Hv-CRKP. The STs and serotypes of 379 strains were marked on the right of the phylogenetic tree. The presence of genes, etc. Is represented by a solid box. And the absence of others is represented by a white box. st, sequence type; ND, not defined; HvKP, hypervirulent *Klebsiella pneumoniae*; CRKP, carbapenem-resistant *Klebsiella pneumoniae*; Hv-CRKP, hypervirulent carbapenem-resistant *Klebsiella pneumoniae*.

SNP-based phylogenetic analysis indicated that genetic divergence between ST258 and ST512 was minimal (<10.0% sequence variation), whereas ST258/ST512 strains exhibited greater genetic distance from ST11 (ranging from 20.0% to 30.0%) (Figure 5). These findings support closer evolutionary relatedness between ST258 and ST512 than between either and ST11.

### Conjugation rates

In the first-round conjugation assays, the conjugation rates of the drug-resistance plasmid pKPHS2 (harbored by HS11286^*recC*2804G^+*arr-3*) to NTUH-K2044, pKPHS2 (from HS11286^*recC*2804A^+*arr-3*) to NTUH-K2044, pKPHS2 (from HS11286^*recC*2804G^+*arr-3*) to NTUH-K2044Δ*wzy-K1*, and pKPHS2 (from HS11286^*recC*2804A^+*arr-3*) to NTUH-K2044Δ*wzy-K1* were (1.85 ± 0.46) × 10^−9^, (2.12 ± 0.34) × 10^−9^, (9.87 ± 1.81) × 10^−9^, and (11.95 ± 3.77) × 10^−9^ (Table S5), respectively.

In the second-round conjugation experiments, the conjugation rates of pK2044 (transferred to HS11286^*recC*2804G^+*arr-3* from NTUH-K2044+pKPHS2), pK2044 (to HS11286^*recC*2804A^+*arr-3* from NTUH-K2044+pKPHS2), pK2044 (to HS11286^*recC*2804G^+*arr-3* from NTUH-K2044Δ*wzy-K1*+pKPHS2), and pK2044 (to HS11286^*recC*2804A^+*arr-3* from NTUH-K2044Δ*wzy-K1*+pKPHS2) were (3.56 ± 2.11) × 10^−12^, (27.67 ± 19.13) × 10^−12^, (4.44 ± 0.79) × 10^−12^, and (15.29 ± 18.77) × 10^−12^ (Table S5), respectively.

These results demonstrated that the RecC^935H^ mutation had no significant effect on pKPHS2 transfer to NTUH-K2044 (average 0.18-fold change) but significantly enhanced pK2044 transfer to HS11286 strains (average 4.61-fold increase). Additionally, deletion of *wzy-K1* enhanced pKPHS2 transfer to NTUH-K2044 (average 4.49-fold increase) but showed no impact on pK2044 transfer to HS11286 (average 0.10-fold change).

### Virulence plasmid retention rates

**Figure 6.**
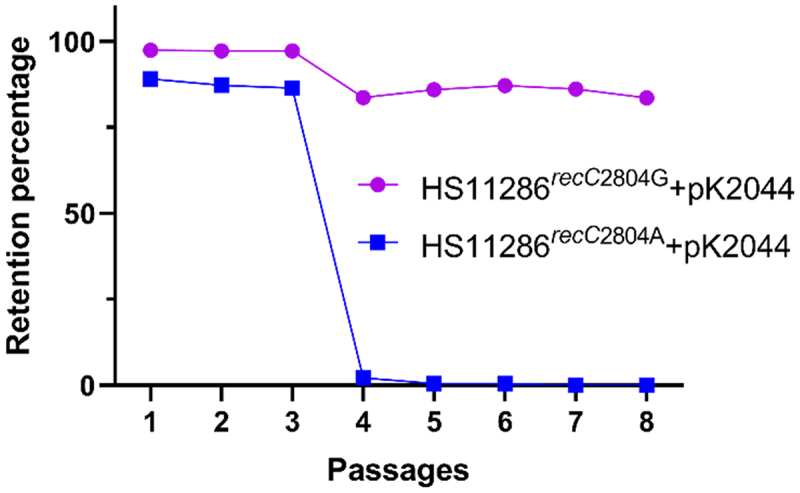
Retention rates of pK2044 in HS11286^*recC*2804G^+*arr-3* and HS11286^*recC*2804A^+*arr-3*. Wilcoxon test was used to compare the retention rates of pK2044 in HS11286^recC2804G^+arr-3 and HS11286^recC2804A^+arr-3 and a *p* value of 0.0078 was yielded.

As shown in Figure 6 and Table S6-S7, the retention rates of pK2044 in HS11286^*recC*2804G^+*arr-3* remained above 95% during the first 3 passages and declined to over 80% in the subsequent 5 passages, with the turning point occurring at the 4th passage. In contrast, the retention rates of pK2044 in HS11286^*recC*2804A^+*arr-3* remained above 85% in the first 3 passages but plummeted to less than 3% in the remaining 5 passages, also with the turning point at the 4th passage.

## Discussion

We sought to elucidate inter-strain variations within the CG258 *K. pneumoniae* strains deposited in GenBank, focusing on serotypes, antibiotic-resistance genes, OMP-related genes, virulence genes, and the RecBCD/A system. Phylogenetic relatedness was assessed via SNP analysis, and preliminary functional impacts of the RecC^935H^ mutation were also investigated.

A total of 79 distinct serotypes were identified *K. pneumoniae* strains [26]. Notably, ST11 strains exhibited nearly no serotype overlap with ST258 or ST512. Most ST512 strains belonged to serotype KL107, which was also prevalent in ST258, suggesting ST512 may represent a close descendant of ST258. Carbapenemase gene (*bla*_KPC_) dominated resistance profiles in all three ST groups (Figure 1(B)). However, subtype distribution differed significantly: ST258 strains carried both *bla*_KPC-2_ and *bla*_KPC-3_; ST11 strains primarily harbored *bla*_KPC-2_; and ST512 strains were enriched for *bla*_KPC-3_. This distribution correlated with geographic patterns: *bla*_KPC-2_ was most common in Chinese isolates, whereas *bla*_KPC-3_ predominated in strains from the United States and Italy (Table S2).

Among OMP-related genes and their regulators, only *kbvR* showed significant variation in prevalence across groups (Figure 1(C)): it was most frequent in ST11 (97.18%), intermediate in ST258 (68.75%), and rarest in ST512 (6.67%). Gene *kbvR* directly regulates *ompK36* expression [31]; its deletion reduces *ompK36* levels, enhancing antimicrobial susceptibility—particularly to β-lactams, including carbapenems. Additionally, *kbvR* contributes to capsule biosynthesis, antiphagocytosis, and virulence in *K. pneumoniae* [32], suggesting its absence may attenuate virulence while potentiating antibiotic resistance in ST258 and ST512.

The only virulence gene with significant inter-group (ST258 and ST512) variation was *irp2* (Figure 1(D)). ST11 strains harbored a broader array of virulence factors than ST258 or ST512, including *peg-344*, *p-rmpA*, *p-rmpA2*, *wza*, *irp2*, *iroN*, and *iucA*—most of which are typically located on pLVPK-like plasmids (except *wza*) [33], indicating a heightened hypervirulence potential. For virulence factors, ST258 strains showed a higher prevalence of 2 siderophores, while ST512 strains had a higher rate of 1 siderophore; ST11 strains exhibited the highest rates of hypercapsules and 2–4 siderophores (Figure 1(E)).

All three ST groups displayed comparable CRKP rates (>86.0%; Figure 2(A)). However, ST11 strains had significantly higher proportions of HvKP and Hv-CRKP than ST258 or ST512 (<3.0% in the latter two groups). Hv-CRKP was first reported in GenBank in 2017 and remains rare in ST258/ST512 but relatively prevalent in ST11. Although Hv-CRKP generally has a clinical advantage over CRKP, its prevalence in ST11 declined notably in 2022 (Figure 2(B))—a trend requiring further investigation. Plasmids were identified as key vectors for antimicrobial resistance genes and virulence genes, largely driving carbapenem resistance and hypervirulence (Figure 2(C–F)), consistent with their role as targets of the *K. pneumoniae* RecBCD/A immune system [34].

The RecBCD/A system, which collaborates with the Restriction-Modification system to facilitate recombination of cleaved nucleotide strands, was analyzed across ST258, ST11, and ST512 strains. Among the four genes (*recA*, *recB*, *recC*, and *recD*) composing this system, only *recC* exhibited notable genetic variation among the three clonal groups (Figure 3(C)). No significant differences were detected in *recA*, *recB*, or *recD* sequences, suggesting high conservation of these components in CG258. Sequence alignment of *recC* revealed two distinct allelic variants: RecC^935R^ (clade II) and RecC^935H^ (clade I). Notably, ST258 and ST512 strains exclusively belonged to clade II (RecC^935R^), which correlated with a near-complete absence of hypervirulence (Figure 3(E)). In contrast, ST11 strains predominantly harbored clade I (RecC^935H^), which was strongly associated with hypervirulence (Figure 3(E)). This suggests that RecC^935H^ may play a critical role in obtaining or retaining virulence plasmids in ST11 strains.

Structural modeling of RecC^935R^ and RecC^935H^ revealed that the amino acid substitution at position 935 (R→H) did not alter the overall conformational structure of the RecC enzyme (Figure 4(A)), except for a localized change in an amino acid side chain. However, detailed analysis uncovered a loss of hydrogen bonding between residue H935 and E890 in RecC^935H^, whereas this bond was preserved in RecC^935R^ (Figures 4(B,C)). Position 935 lies within the hinge region of the nucleotide recognition domain [35], suggesting that the loss of this hydrogen bond may upregulate nucleotide insertion kinetics, thereby enhancing recombination efficiency [36]. Given the earlier emergence of CRKP compared to Hv-CRKP, it is hypothesized that RecC^935H^ likely arose from RecC^935R^ variants through evolutionary adaptation.

SNP-based phylogenetic analysis (Figure 5) provided two key insights: (i) ST258 and ST512 strains are more closely related to each other than to ST11; (ii) ST11 exhibits greater evolutionary divergence from ST258/ST512, indicating more active genetic evolution. Despite Hv-CRKP’s competitive advantage over CRKP and HvKP (Figure 2(B)), its prevalence in *K. pneumoniae* populations has not increased continuously, a trend warranting further investigation. Notably, while ST258 and ST512 are often described as “immune-deficient” [37,38], they demonstrated superior competence in horizontal gene transfer compared to ST11 strains.

Collectively, these findings suggest that RecC^935H^ may drive the dominance of hypervirulence traits in ST11 strains, and its increasing prevalence (consistent with recent reports [36]) could shape the future evolutionary trajectory of ST11 *K. pneumoniae*.

Conjugation assays confirmed that the RecC^935H^ mutation had no significant impact on the transfer efficiency of pKPHS2 to NTUH-K2044 (average 0.18-fold change), but significantly facilitated the transfer of pK2044 to HS11286 strains (average 4.61-fold enhancement). Conversely, deletion of *wzy-K1* enhanced pKPHS2 transfer to NTUH-K2044 (average 4.49-fold increase) but showed no advantage for pK2044 transfer to HS11286 (average 0.10-fold change). Since most virulence plasmids are not self-transmissible and require assistance from mobilizable plasmids (e.g. IncFII plasmids carrying resistance genes) [39], this facilitation highlights the adaptive advantage of virulence plasmids in transferring to CRKP. Virulence plasmids are primarily derived from hypervirulent *K. pneumoniae*, particularly K1 and K2 strains. Notably, the majority of CRKP strains harboring virulence plasmids do not exhibit hypervirulence [40], consistent with the absence of hypercapsules [30,41]. Our experimental models—NTUH-K2044 (hypercapsule) and NTUH-K2044Δ*wzy-K1* (slim capsule) [30]—thus mimicked real-world scenarios of virulence plasmid transfer. RecC^R935H^ mutation together with slim capsules enhanced the transfer of virulence plasmids into CRKP (29.8-fold increase).

Unexpectedly, RecC^935H^ accelerated the loss of virulence plasmids in CRKP strains (Figure 6). When considering the first-generation isolation (24 hours), virulence plasmids in HS11286^*recC*2804A^+*arr-3* were nearly undetectable within 3 days, representing a substantial fitness cost. Notably, 3 days correspond to over 72 bacterial generations. Collectively, the RecC^935H^ mutation enhances the transfer of virulence plasmids into CRKP but reduces their long-term retention.

This study has two main limitations. First, due to the distinct regional distribution of ST258/ST512 and ST11 strains, collecting sufficient representative isolates was challenging. Second, strains in GenBank could have been influenced by the propensity of researchers worldwide and do not inevitably reflect the exact clinical distribution.

## Conclusions

In summary, despite belonging to the same CG258 group, ST258, ST11, and ST512 *K. pneumoniae* strains exhibit significant differences in serotypes, antibiotic-resistance genes, virulence genes, and the RecBCD/A system. While all three groups share carbapenem resistance driven by *bla*_KPC_ variants (e.g. *bla*_KPC-2_ and *bla*_KPC-3_), ST11 strains display a markedly higher propensity for hypervirulence. This trait is associated with the RecC^935H^ mutation, which facilitates the transfer of virulence plasmids into CRKP strains, underscoring its role in shaping the evolution of Hv-CRKP.

## Supplementary Material

Supplement legend.docx

Change of Authorship Request Form.docx

## Data Availability

The datasets supporting the conclusions of this article along with the supplementary tables are available in Science Data Bank (https://doi.org/10.57760/sciencedb.11972) [].

## References

[1] Choby JE, Howard-Anderson J, Weiss DS. Hypervirulent Klebsiella pneumoniae - clinical and molecular perspectives. J Intern Med. 2020;287(3):283–14. doi: 10.1111/joim.13007 31677303 PMC7057273

[2] Chen IR, Lin S-N, Wu X-N, et al. Clinical and microbiological characteristics of bacteremic pneumonia caused by Klebsiella pneumoniae. Front Cell Infect Microbiol. 2022;12:903682. doi: 10.3389/fcimb.2022.903682 35811668 PMC9259976

[3] Dong N, Yang X, Chan EW-C, et al. Klebsiella species: taxonomy, hypervirulence and multidrug resistance. EBioMedicine. 2022;79:103998. doi: 10.1016/j.ebiom.2022.103998 35405387 PMC9010751

[4] Wyres KL, Holt KE. Klebsiella pneumoniae population genomics and antimicrobial-resistant clones. Trends Microbiol. 2016;24(12):944–956. doi: 10.1016/j.tim.2016.09.007 27742466

[5] Yoshioka N, Hagiya H, Deguchi M, et al. Multiplex real-time PCR assay for six major carbapenemase genes. Pathogens. 2021;10(3):276. doi: 10.3390/pathogens10030276 33804402 PMC7999841

[6] Zhang Y, Zeng J, Liu W, et al. Emergence of a hypervirulent carbapenem-resistant Klebsiella pneumoniae isolate from clinical infections in China. J Infect. 2015;71(5):553–560. doi: 10.1016/j.jinf.2015.07.010 26304687

[7] Pages JM, James CE, Winterhalter M. The porin and the permeating antibiotic: a selective diffusion barrier in Gram-negative bacteria. Nat Rev Microbiol. 2008;6(12):893–903. doi: 10.1038/nrmicro1994 18997824

[8] Brunson DN, Maldosevic E, Velez A, et al. Porin loss in Klebsiella pneumoniae clinical isolates impacts production of virulence factors and survival within macrophages. Int J Med Microbiol. 2019;309(3–4):213–224. doi: 10.1016/j.ijmm.2019.04.001 31010630

[9] Fang CT, Chuang Y-P, Shun C-T, et al. A novel virulence gene in Klebsiella pneumoniae strains causing primary liver abscess and septic metastatic complications. J Exp Med. 2004;199(5):697–705. doi: 10.1084/jem.20030857 14993253 PMC2213305

[10] Kuo-Ming Y, Lin J-C, Yin F-Y, et al. Revisiting the importance of virulence determinant magA and its surrounding genes in Klebsiella pneumoniae causing pyogenic liver abscesses: exact role in serotype K1 capsule formation. J Infect Dis. 2010;201(8):1259–1267. doi: 10.1086/606010 19785524

[11] Li D, Huang X, Rao H, et al. Klebsiella pneumoniae bacteremia mortality: a systematic review and meta-analysis. Front Cell Infect Microbiol. 2023;13:1157010. doi: 10.3389/fcimb.2023.1157010 37153146 PMC10159367

[12] Zhang Y, Jin L, Ouyang P, et al. Evolution of hypervirulence in carbapenem-resistant Klebsiella pneumoniae in China: a multicentre, molecular epidemiological analysis. J Antimicrob Chemother. 2020;75(2):327–336. doi: 10.1093/jac/dkz446 31713615

[13] Xu L, Sun X, Ma X. Systematic review and meta-analysis of mortality of patients infected with carbapenem-resistant Klebsiella pneumoniae. Ann Clin Microbiol Antimicrob. 2017;16(1):18. doi: 10.1186/s12941-017-0191-3 28356109 PMC5371217

[14] Huang N, Jia H, Zhou B, et al. Hypervirulent carbapenem-resistant Klebsiella pneumoniae causing highly fatal meningitis in southeastern China. Front Public Health. 2022;10:991306. doi: 10.3389/fpubh.2022.991306 36324461 PMC9621088

[15] Li Y, Hu D, Ma X, et al. Convergence of carbapenem resistance and hypervirulence leads to high mortality in patients with postoperative Klebsiella pneumoniae meningitis. J Glob Antimicrob Resist. 2021;27:95–100. doi: 10.1016/j.jgar.2021.02.035 34133987

[16] Gu D, Dong N, Zheng Z, et al. A fatal outbreak of ST11 carbapenem-resistant hypervirulent Klebsiella pneumoniae in a Chinese hospital: a molecular epidemiological study. Lancet Infect Dis. 2018;18(1):37–46. doi: 10.1016/S1473-3099(17)30489-9 28864030

[17] Teo JQ, Tang CY, Tan SH, et al. Genomic surveillance of carbapenem-resistant Klebsiella pneumoniae from a major public health hospital in Singapore. Microbiol Spectr. 2022;10(5):e0095722. doi: 10.1128/spectrum.00957-22 36066252 PMC9602435

[18] Qi Y, Wei Z, Ji S, et al. St11, the dominant clone of KPC-producing Klebsiella pneumoniae in China. J Antimicrob Chemother. 2011;66(2):307–312. doi: 10.1093/jac/dkq431 21131324

[19] Mathers AJ, Peirano G, Pitout JD. The role of epidemic resistance plasmids and international high-risk clones in the spread of multidrug-resistant Enterobacteriaceae. Clin Microbiol Rev. 2015;28(3):565–591. doi: 10.1128/CMR.00116-14 25926236 PMC4405625

[20] Di Pilato V, Errico G, Monaco M, et al. The changing epidemiology of carbapenemase-producing Klebsiella pneumoniae in Italy: toward polyclonal evolution with emergence of high-risk lineages. J Antimicrob Chemother. 2021;76(2):355–361. doi: 10.1093/jac/dkaa431 33188415

[21] Luterbach CL, Chen L, Komarow L, et al. Transmission of carbapenem-resistant Klebsiella pneumoniae in US hospitals. Clin Infect Dis. 2023;76(2):229–237. doi: 10.1093/cid/ciac791 36173830 PMC10202433

[22] Wang Q, Wang X, Wang J, et al. Phenotypic and genotypic characterization of carbapenem-resistant enterobacteriaceae: data from a longitudinal large-scale CRE study in China (2012–2016). Clin Infect Dis. 2018;67(suppl_2):S196–S205. doi: 10.1093/cid/ciy660 30423057

[23] Yu F, Lv J, Niu S, et al. Multiplex PCR analysis for rapid detection of Klebsiella pneumoniae carbapenem-resistant (sequence type 258 [ST258] and ST11) and hypervirulent (ST23, ST65, ST86, and ST375) strains. J Clin Microbiol. 2018;56(9). doi: 10.1128/JCM.00731-18 PMC6113471 29925644

[24] Lam MMC, Wyres KL, Duchêne S, et al. Population genomics of hypervirulent Klebsiella pneumoniae clonal-group 23 reveals early emergence and rapid global dissemination. Nat Commun. 2018;9(1):2703. doi: 10.1038/s41467-018-05114-7 30006589 PMC6045662

[25] Hu D, Li Y, Ren P, et al. Molecular epidemiology of hypervirulent carbapenemase-producing Klebsiella pneumoniae. Front Cell Infect Microbiol. 2021;11:661218. doi: 10.3389/fcimb.2021.661218 33898334 PMC8058458

[26] Paczosa MK, Mecsas J. Klebsiella pneumoniae: going on the offense with a strong defense. Microbiol Mol Biol Rev. 2016;80(3):629–661. doi: 10.1128/MMBR.00078-15 27307579 PMC4981674

[27] Tamura K, Stecher G, Kumar S. Mega11: molecular evolutionary genetics analysis version 11. Mol Biol Evol. 2021;38(7):3022–3027. doi: 10.1093/molbev/msab120 33892491 PMC8233496

[28] Gardner SN, Slezak T, Hall BG. kSNP3.0: sNP detection and phylogenetic analysis of genomes without genome alignment or reference genome. Bioinformatics. 2015;31(17):2877–2878. doi: 10.1093/bioinformatics/btv271 25913206

[29] Datsenko KA, Wanner BL. One-step inactivation of chromosomal genes in Escherichia coli K-12 using PCR products. Proc Natl Acad Sci USA. 2000;97(12):6640–6645. doi: 10.1073/pnas.120163297 10829079 PMC18686

[30] Hu D, Chen W, Wang W, et al. Hypercapsule is the cornerstone of Klebsiella pneumoniae in inducing pyogenic liver abscess. Front Cell Infect Microbiol. 2023;13:1147855. doi: 10.3389/fcimb.2023.1147855 37065211 PMC10102340

[31] Wang M, Tian Y, Xu L, et al. High osmotic stress increases OmpK36 expression through the regulation of KbvR to decrease the antimicrobial resistance of Klebsiella pneumoniae. Microbiol Spectr. 2022;10(3):e0050722. doi: 10.1128/spectrum.00507-22 35658577 PMC9241633

[32] Xu L, Wang M, Yuan J, et al. The KbvR regulator contributes to capsule production, outer membrane protein biosynthesis, antiphagocytosis, and virulence in Klebsiella pneumoniae. Infect Immun. 2021;89(5). doi: 10.1128/IAI.00016-21 PMC8091090 33593891

[33] Dai P, Hu D. The making of hypervirulent Klebsiella pneumoniae. J Clin Lab Anal. 2022;36(12):e24743. doi: 10.1002/jcla.24743 36347819 PMC9757020

[34] Dimitriu T, Szczelkun MD, Westra ER. Evolutionary ecology and interplay of prokaryotic innate and adaptive immune systems. Curr Biol. 2020;30(19):R1189–R1202. doi: 10.1016/j.cub.2020.08.028 33022264 PMC7116224

[35] Amundsen SK, Smith GR. Recbcd enzyme: mechanistic insights from mutants of a complex helicase-nuclease. Microbiol Mol Biol Rev. 2023;87(4):e0004123. doi: 10.1128/mmbr.00041-23 38047637 PMC10732027

[36] Zhou K, Xue C-X, Xu T, et al. A point mutation in recC associated with subclonal replacement of carbapenem-resistant Klebsiella pneumoniae ST11 in China. Nat Commun. 2023;14(1):2464. doi: 10.1038/s41467-023-38061-z 37117217 PMC10147710

[37] Zhou Y, Tang Y, Fu P, et al. The type I-E CRISPR-Cas system influences the acquisition of bla(KPC)-IncF plasmid in Klebsiella pneumoniae. Emerg Microbes Infect. 2020;9(1):1011–1022. doi: 10.1080/22221751.2020.1763209 32393110 PMC7301723

[38] Zhou Y, Tian D, Tang Y, et al. High-risk KPC-producing Klebsiella pneumoniae lack type I R-M systems. Int J Antimicrob Agents. 2020;56(2):106050. doi: 10.1016/j.ijantimicag.2020.106050 32544567

[39] Tian D, Liu X, Chen W, et al. Prevalence of hypervirulent and carbapenem-resistant Klebsiella pneumoniae under divergent evolutionary patterns. Emerg Microbes Infect. 2022;11(1):1–42. doi: 10.1080/22221751.2022.2103454 35844192 PMC9359173

[40] Yang X, Sun Q, Li J, et al. Molecular epidemiology of carbapenem-resistant hypervirulent Klebsiella pneumoniae in China. Emerg Microbes Infect. 2022;11(1):841–849. doi: 10.1080/22221751.2022.2049458 35236251 PMC8942559

[41] Xu L, Li J, Wu W, et al. Klebsiella pneumoniae capsular polysaccharide: mechanism in regulation of synthesis, virulence, and pathogenicity. Virulence. 2024;15(1):2439509. doi: 10.1080/21505594.2024.2439509 39668724 PMC11649230

