# Supplementary material for: RecC935H is associated with divergent evolutions of clonal group 258 Klebsiella pneumoniae
Source: Virulence. 2026 May 13;17(1):2673646. doi: 10.1080/21505594.2026.2673646 (PMC13192128; doi:10.1080/21505594.2026.2673646)
Supplement: Supplement legend.docx [file KVIR_A_2673646_SM0792.docx]

**RecC^935H^ is associated with Divergent Evolutions of Clonal Group 258 *Klebsiella pneumoniae***

**Running title: RecC^935H^ is with Evolutions of *Klebsiella pneumoniae***

Dakang Hu^1†^, Xinru Ye ^2†^, Jie Wang^3†^, Shuli Mi^2^, Jiawen Sun^2^, Piaopiao Dai^1^, Tingting Huang^4^, Jin Zhang^1^, Xinhua Luo^1^, Qinfei Ma^1^, Xiaofei Jiang^5^, Susu Wu^1,^ ^6*^, Haifang Zhang^6,7,8*^

^1. Department of Laboratory Medicine, Taizhou Municipal Hospital (Taizhou University Affiliated Municipal Hospital), School of Medicine, Taizhou University, Taizhou 318000, Zhejiang, China.^

^2. Taizhou University, Taizhou318000, Zhejiang, China.^

^3. VIP ward, Huashan Hospital, Fudan University, Shanghai 200040, China.^

^4. Department of Medical Laboratory, Taizhou Traditional Chinese Medicine Hospital, Taizhou 318000, Zhejiang, China.^

^5. Department of Medical Laboratory, Huashan Hospital, Fudan University, Shanghai 200040, China.^

^6. Department of Clinical Laboratory, The Second Affiliated Hospital of Soochow University, Suzhou 215004, Jiangsu, China^

^7. MOE Key Laboratory of Geriatric Diseases and Immunology, Soochow University, Suzhou 215004, Jiangsu, China^

^8. Lead contact^

† These authors contributed equally to this work

*: Corresponding authors:

Susu Wu, MM, Department of Laboratory Medicine, Taizhou Municipal Hospital (Taizhou University Affiliated Municipal Hospital), School of Medicine, Taizhou University, Taizhou 318000, Zhejiang, China; Department of Clinical Laboratory, The Second Affiliated Hospital of Soochow University, Suzhou 215004, Jiangsu, China. E-mail: wususu84@163.com. Tel: 86-576-88858142. Fax: 86-576-88858284.

Haifang Zhang, PhD, Department of Clinical Laboratory, Second Affiliated Hospital of Soochow University, Suzhou 215004, China; MOE Key Laboratory of Geriatric Diseases and Immunology, Soochow University, Suzhou 215004, Jiangsu, China; Lead contact. E-mail: haifangzhang@suda.edu.cn. Tel: 86-512-67783550. Fax: 86-512-67783550.

**Abstract**

Clonal group 258 *Klebsiella pneumoniae* strains are notorious but their divergent evolutions were identified through analysis of 80 sequence type (ST) 258, 284 ST11, and 15 ST512 genomes. The serotypes of ST11 strains differed from those of ST258 and ST512. Genes *bla*_KPC-2_ and *bla*_KPC-3_ dominated carbapenem-resistance in ST258 strains, whereas they were found in ST11 and ST512, respectively. The carbapenem-resistance rates were 93.75%, 86.97%, and 100.0% among the ST258, ST11, and ST512 strains, respectively while the hypervirulence and carbapenem-resistance rates were 2.50%, 35.92%, and 0.00%, respectively. The wide carbapenem-resistance and the hypervirulence were determined by drug-resistance and virulence plasmids respectively. Except *recA*, *recB*, and *recD*, *recC* was significantly different: RecC^935R^ for the three groups and RecC^935H^ for ST11; RecC^935H^ ST11 strains presented a higher rate of hypervirulence plus carbapenem-resistance than those carrying RecC^935R^. RecC^935H^ and RecC^935R^ presented the difference of an amino acid side chain, leading to the disappearance of the hydrogen bond. Single-Nucleotide Polymorphism analysis verified closer relationship between ST258 and ST512 than ST11. RecC^R935H^ facilitated pK2044 to HS11286 (5.61 times) while *wzy-K1* deletion exerted advantages on pKPHS2 to NTUH-K2044 (5.49 times). The retention rates of pK2044 in HS11286*^recC^*^2804G^+*arr-3* kept over 80.0% in the 8 passages while those in HS11286*^recC^*^2804A^+*arr-3* declined to less than 3.0% in the last 5 passages. In conclusion, different from ST258 and ST512, ST11 strains show an overwhelming propensity to become hypervirulent and carbapenem-resistant. RecC^R935H^ mutation facilitates the transfer of virulence plasmids into carbapenem-resistant *K. pneumoniae* strains but decreases their retention in the strains.

**Keywords:** *Klebsiella pneumoniae*; Drug resistance; Virulence; Sequence type; Evolution

**Supplement legends:**

Table S1 1407 *Klebsiella pneumoniae* genomes from GenBank

Notes: ST: sequence type; ND: not determined.

Table S2 Traits of 379 clonal group 258 genomes

Notes: ST: sequence type; bp: base pair; Omp: outer membrane protein; Bla: beta lactamase; Numbers and abscence in the brackets (under Omp-related genes, Bla genes, and virulence genes) mean positive and negative genes respectively.

Table S3 Outer membrane protein-related nucleotide sequences of SGH-10

Table S4 Primers used in the study

Note: a: high-fidelity PCR; b: regular PCR.

Table S5 Colony forming units in conjutaion assays

Table S6 Colony forming units in virulence plasmid retention assays (HS11286*^recC^*^2804G^+*arr-3*+pK2044)

Note: non-selective LBAP: Luria-Bertani agar plate; selective LBAP: Luria-Bertani agar plate supplemented with 1.5 μg/mL potassium tellurite and 2.0 μg/mL meropenem.

Table S7 Colony forming units in virulence plasmid retention assays (HS11286*^recC^*^2804A^+*arr-3*+pK2044)

Note: non-selective LBAP: Luria-Bertani agar plate; selective LBAP: Luria-Bertani agar plate supplemented with 1.5 μg/mL potassium tellurite and 2.0 μg/mL meropenem.
