# Supplementary material for: RecC935H is associated with divergent evolutions of clonal group 258 Klebsiella pneumoniae
Source: Virulence. 2026 May 13;17(1):2673646. doi: 10.1080/21505594.2026.2673646 (PMC13192128; doi:10.1080/21505594.2026.2673646)
Supplement: Change of Authorship Request Form.docx [file KVIR_A_2673646_SM0791.docx]

**Change of authorship request form – Journals**

*Please complete this form fully; if it is not completed, it will not be reviewed and returned to you. Please note that if an authorship change is made for a journal that does not consider authorship changes, your submission may be automatically withdrawn and returned to you.*

**Where appropriate, the journal reserves the right to publish the authorship history of the article, including all previous author lists; by completing this form, authors grant permission for this information to be included in the published article.**

**If a request is made which is considered in breach of the journal’s editorial policies, the journal reserves the right to inform the relevant authors’ institution.**

| **Title of manuscript:** | **RecC^935H^ is associated with Divergent Evolutions of Clonal Group 258 *Klebsiella pneumoniae*** |
| --- | --- |
| **Manuscript ID no.:** | **240615688** |
| **Journal Name:** | **Virulence** |
| **Total no. of authors added:** | **0** |
| **Total no. of authors removed:** | **0** |

1. **Original author list at submission, in the order shown on the manuscript. Please indicate the corresponding author with a ***

| **Original author list at submission** | **First name(s)** | **Family name** | **Affiliation (institute/organisation, department)** | **Email address** |
| --- | --- | --- | --- | --- |
| **1^st^ author** | **Susu** | **Wu** | ^1. Department of Clinical Laboratory, The Second Affiliated Hospital of Soochow University^  ^2. Department of Laboratory Medicine, Taizhou Municipal Hospital^ | wususu84@yeah.net |
| **2^nd^ author** | **Xinru** | **Ye** | ^Taizhou University^ | 1216400557@qq.com |
| **3^rd^ author** | **Jie** | **Wang** | ^VIP ward, Huashan Hospital, Fudan University^ | 327210710@qq.com |
| **4^th^ author** | **Shuli** | **Mi** | ^Taizhou University^ | 1950108879@qq.com |
| **5^th^ author** | **Jiawen** | **Sun** | ^Taizhou University^ | 1161026650@qq.com |
| **6^th^ author** | **Piaopiao** | **Dai** | ^Department of Laboratory Medicine, Taizhou Municipal Hospital^ | dpp0330@126.com |
| **7^th^ author** | **Tingting** | **Huang** | ^Department of Medical Laboratory, Taizhou Traditional Chinese Medicine Hospital^ | 1725880426@qq.com |
| **8^th^ author** | **Jin** | **Zhang** | ^Department of Laboratory Medicine, Taizhou Municipal Hospital^ | zhangjin_jhb@163.com |
| **9^th^ author** | **Xinhua** | **Luo** | ^Department of Laboratory Medicine, Taizhou Municipal Hospital^ | luoxiangnanchang84@163.com |
| **10^th^ author** | **Qinfei** | **Ma** | ^Department of Laboratory Medicine, Taizhou Municipal Hospital^ | 13957687070@163.com |
| **11^th^ author** | **Dakang** | **Hu** | ^Department of Laboratory Medicine, Taizhou Municipal Hospital^ | 18111220048@fudan.edu.cn |
| **12^th^ author** | **Xiaofei** | **Jiang** | ^Department of Medical Laboratory, Huashan Hospital, Fudan University^ | jiangxi2154@sina.com |
| **13^th^ author** | **Haifang** | **Zhang** | ^Department of Clinical Laboratory, The Second Affiliated Hospital of Soochow University^ | haifangzhang@suda.edu.cn |

*Please insert new rows if needed*

1. Please provide an explanation for the change in authorship **(including any reasons for additions to, or removals from the original authorship list)**.
2. Dakang Hu did the functional experiments between 2025.02 and 2025.07 and is suggested to be the first author. His roles included conceptulization, formal analysis, funding acquisition, resources, investigation, methodology, project administration, validation, and writing-review&editing.
3. The roles of Susu Wu included conceptulization, data curation, formal analysis, funding acquisition, resources, and writing-original draft and is suggested to be the equal corresponding author.
4. Xiaofei Jiang is removed from correspondence authors in line with the instructions of Virulence.
5. Two fundings were added: Zhejiang Provincial Health Commission (Recipient: Susu Wu; Grant number: 2025KY462.) and the Discipline Construction of the Second Affiliated Hospital of Soochow University (Recipient: Haifang Zhang; Grant number: XKTJ-TD2024003) since they are involved in the study. Four fundings were removed: the Gusu Health Youth Talent of Suzhou (grant numbers GSWS2019039 and GSWS2020030); the Science and Technology Program of Suzhou (grant number SKY2021007); and the Discipline Construction of the Second Affiliated Hospital of Soochow University (grant number XKTJ-TD202001) since they all expired.
6. The affiliation of Haifang Zhang is added because he joined in it after the manuscript was submitted last year.
7. **Proposed new authorship list on the current submission (including email addresses), in the order it should appear on the manuscript. Please indicate the corresponding author with an asterix (*).**

| **Proposed new author list and order** | **First name(s)** | **Family name** | **Affiliation (institution/organisation)** | **Email address** | **Please provide details of how this author contributed to the article & the reported findings.** |
| --- | --- | --- | --- | --- | --- |
| **1^st^ author** | **Dakang** | **Hu** | ^Department of Laboratory Medicine, Taizhou Municipal Hospital (Taizhou University Affiliated Municipal Hospital), School of Medicine, Taizhou University^ | 18111220048@fudan.edu.cn | conceptulization, formal analysis, funding acquisition, resources, investigation, methodology,  project administration, validation, writing-review&editing |
| **2^nd^ author** | **Xinru** | **Ye** | ^Taizhou University^ | 1216400557@qq.com | data curation, formal analysis, resources,  writing-original draft |
| **3^rd^ author** | **Jie** | **Wang** | ^VIP ward, Huashan Hospital, Fudan University^ | 327210710@qq.com | conceptulization, data curation, resources,  writing-original draft |
| **4^th^ author** | **Shuli** | **Mi** | ^Taizhou University^ | 1950108879@qq.com | data curation, resources, |
| **5^th^ author** | **Jiawen** | **Sun** | ^Taizhou University^ | 1161026650@qq.com | data curation, resources, |
| **6^th^ author** | **Piaopiao** | **Dai** | ^Department of Laboratory Medicine, Taizhou Municipal Hospital (Taizhou University Affiliated Municipal Hospital), School of Medicine, Taizhou University^ | dpp0330@126.com | data curation, resources, |
| **7^th^ author** | **Tingting** | **Huang** | ^Department of Medical Laboratory, Taizhou Traditional Chinese Medicine Hospital^ | 1725880426@qq.com | data curation, resources, |
| **8^th^ author** | **Jin** | **Zhang** | ^Department of Laboratory Medicine, Taizhou Municipal Hospital (Taizhou University Affiliated Municipal Hospital), School of Medicine, Taizhou University^ | zhangjin_jhb@163.com | formal analysis |
| **9^th^ author** | **Xinhua** | **Luo** | ^Department of Laboratory Medicine, Taizhou Municipal Hospital (Taizhou University Affiliated Municipal Hospital), School of Medicine, Taizhou University^ | luoxiangnanchang84@163.com | formal analysis |
| **10^th^ author** | **Qinfei** | **Ma** | ^Department of Laboratory Medicine, Taizhou Municipal Hospital (Taizhou University Affiliated Municipal Hospital), School of Medicine, Taizhou University^ | 13957687070@163.com | formal analysis |
| **11^th^ author** | **Xiaofei** | **Jiang** | ^Department of Medical Laboratory, Huashan Hospital, Fudan University^ | jiangxi2154@sina.com | funding acquisition,  writing-review&editing |
| **12^th^ author** | **Susu** | **Wu** | ^1. Department of Laboratory Medicine, Taizhou Municipal Hospital (Taizhou University Affiliated Municipal Hospital), School of Medicine, Taizhou University^  ^2. Department of Clinical Laboratory, The Second Affiliated Hospital of Soochow University^ | wususu84@yeah.net | conceptulization, data curation, formal analysis, funding acquisition, resources,  writing-original draft |
| **13^th^ author** | **Haifang** | **Zhang** | ^1.Department of Clinical Laboratory, The Second Affiliated Hospital of Soochow University^  ^2. MOE Key Laboratory of Geriatric Diseases and Immunology, Soochow University, Suzhou 215004, Jiangsu, China^  ^3. Lead contact^ | haifangzhang@suda.edu.cn | funding acquisition, project administration, writing-review&editing |

*Please insert further rows if required*

1. **All authors (unchanged, added and/or removed) must sign this declaration.**

Signatures can be in the form of Docusign, electronic certified signature, or handwritten signatures can be returned as an image file.

Typed names in the signature box **WILL NOT** be accepted unless accompanied by an additional email confirmation from that co-author agreeing to the changed author list and the explanation of changes outlined in Section 2. This email must come from the same email address assigned to that co-author in the above sections.

| **Author name (first name, last name)** | **Declaration** | **I consent to being named in the acknowledgments if the authorship change is rejected** | **Signature (if typed please ensure confirmation via email is attached)** | **Date signed** |
| --- | --- | --- | --- | --- |
| **Dakang Hu** | **I agree to the new authorship list and contributions shown above in section 3, for the reasons outlined in section 2.** |  | **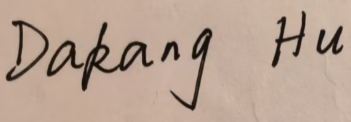** | **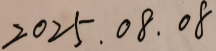** |
| **Xinru Ye** | **I agree to the new authorship list and contributions shown above in section 3, for the reasons outlined in section 2.** |  | **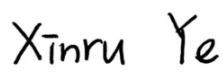** | **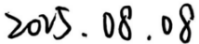** |
| **Jie Wang** | **I agree to the new authorship list and contributions shown above in section 3, for the reasons outlined in section 2.** |  | **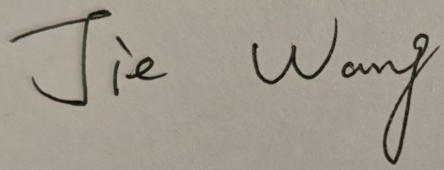** | **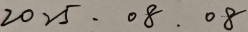** |
| **Shuli Mi** | **I agree to the new authorship list and contributions shown above in section 3, for the reasons outlined in section 2.** |  | **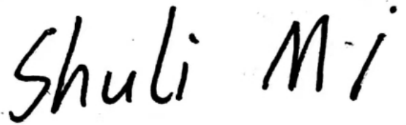** | **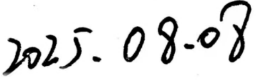** |
| **Jiawen Sun** | **I agree to the new authorship list and contributions shown above in section 3, for the reasons outlined in section 2.** |  | **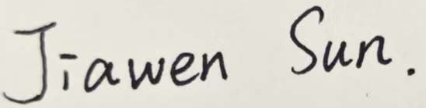** | **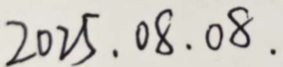** |
| **Piaopiao Dai** | **I agree to the new authorship list and contributions shown above in section 3, for the reasons outlined in section 2.** |  | **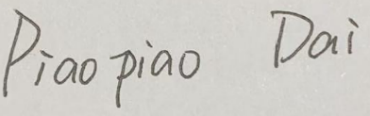** | **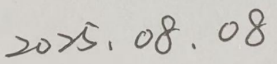** |
| **Tingting Huang** | **I agree to the new authorship list and contributions shown above in section 3, for the reasons outlined in section 2.** |  | **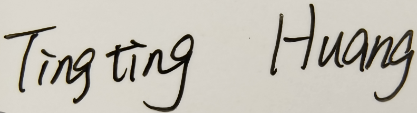** | **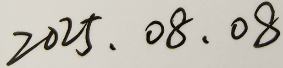** |
| **Jin Zhang** | **I agree to the new authorship list and contributions shown above in section 3, for the reasons outlined in section 2.** |  | **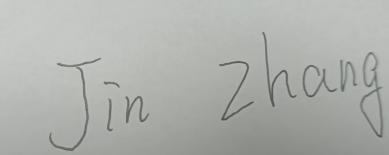** | **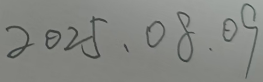** |
| **Xinhua Luo** | **I agree to the new authorship list and contributions shown above in section 3, for the reasons outlined in section 2.** |  | **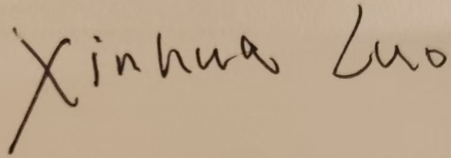** | **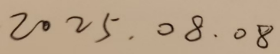** |
| **Qinfei Ma** | **I agree to the new authorship list and contributions shown above in section 3, for the reasons outlined in section 2.** |  | **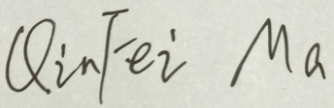** | **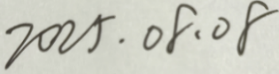** |
| **Xiaofei Jiang** | **I agree to the new authorship list and contributions shown above in section 3, for the reasons outlined in section 2.** |  | **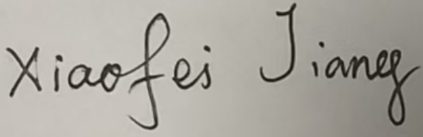** | **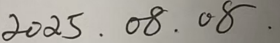** |
| **Susu Wu** | **I agree to the new authorship list and contributions shown above in section 3, for the reasons outlined in section 2.** |  | **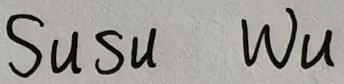** | **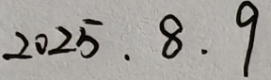** |
| **Haifang Zhang** | **I agree to the new authorship list and contributions shown above in section 3, for the reasons outlined in section 2.** |  | **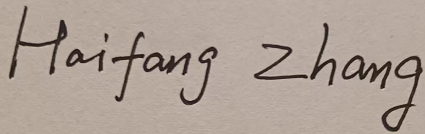** | **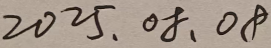** |

*For submissions to journals using the CRediT role taxonomy, please also complete the next page. If the journal uses the CRediT role taxonomy, this will be clearly stated on the journal homepage.*

1. **CRediT roles (if applicable) for proposed author list (please tick the appropriate roles).** For more information on Credit, please consult: <https://credit.niso.org/contributor-roles-defined/>**.**

|  | | **Conceptualization** | **Data Curation** | **Formal analysis** | **Funding acquisition** | **Investigation** | **Methodology** | **Project administration** | **Resources** | **Software** | **Supervision** | **Validation** | **Visualization** | **Writing- original draft** | **Writing- review & editing** |
| --- | --- | --- | --- | --- | --- | --- | --- | --- | --- | --- | --- | --- | --- | --- | --- |
| **Author names** | **Dakang Hu** | √ |  | √ | √ | √ | √ | √ | √ |  |  | √ |  |  | √ |
|  | **Xinru Ye** |  | √ | √ |  |  |  |  | √ |  |  |  |  | √ |  |
|  | **Jie Wang** | √ | √ |  |  |  |  |  | √ |  |  |  |  | √ |  |
|  | **Shuli Mi** |  | √ |  |  |  |  |  | √ |  |  |  |  |  |  |
|  | **Jiawen Sun** |  | √ |  |  |  |  |  | √ |  |  |  |  |  |  |
|  | **Piaopiao Dai** |  | √ |  |  |  |  |  | √ |  |  |  |  |  |  |
|  | **Tingting Huang** |  | √ |  |  |  |  |  | √ |  |  |  |  |  |  |
|  | **Jin Zhang** |  |  | √ |  |  |  |  |  |  |  |  |  |  |  |
|  | **Xinhua Luo** |  |  | √ |  |  |  |  |  |  |  |  |  |  |  |
|  | **Qinfei Ma** |  |  | √ |  |  |  |  |  |  |  |  |  |  |  |
|  | **Xiaofei Jiang** |  |  |  | √ |  |  |  |  |  |  |  |  |  | √ |
|  | **Susu Wu** | √ | √ | √ | √ |  |  |  | √ |  |  |  |  | √ |  |
|  | **Haifang Zhang** |  |  |  | √ |  |  | √ |  |  |  |  |  |  | √ |
